# Guidelines for reporting pediatric and child health clinical trial protocols and reports: study protocol for *SPIRIT-Children* and *CONSORT-Children*

**DOI:** 10.1186/s13063-024-07948-7

**Published:** 2024-01-30

**Authors:** Ami Baba, Maureen Smith, Beth K. Potter, An-Wen Chan, David Moher, Martin Offringa

**Affiliations:** 1 Child Health Evaluative Sciences, The Hospital for Sick Children, Toronto, ON Canada; 2 Patient Partner, Canadian Organization for Rare Disorders, Ottawa, ON Canada; 3 School of Epidemiology and Public Health, University of Ottawa, Ottawa, ON Canada; 4 Department of Medicine, Women’s College Research Institute, University of Toronto, Toronto, ON Canada; 5 Centre for Journalology, Clinical Epidemiology Program, Ottawa Hospital Research Institute, Ottawa, ON Canada; 6 Institute of Health Policy, Management and Evaluation, University of Toronto, Toronto, ON Canada; 7 Peter Gilgan Centre for Research and Learning, The Hospital for Sick Children, 686 Bay Street, Toronto, ON M5G 0A4 Canada

**Keywords:** SPIRIT, CONSORT, Reporting guideline, Clinical trial, Trial protocol, Trial report, Pediatrics, Young people, Child health, youth involvement, Patient and public involvement

## Abstract

**Background:**

Despite the critical importance of clinical trials to provide evidence about the effects of intervention for children and youth, a paucity of published high-quality pediatric clinical trials persists. Sub-optimal reporting of key trial elements necessary to critically appraise and synthesize findings is prevalent. To harmonize and provide guidance for reporting in pediatric controlled clinical trial protocols and reports, reporting guideline extensions to the Standard Protocol Items: Recommendations for Interventional Trials (SPIRIT) and Consolidated Standards of Reporting Trials (CONSORT) guidelines specific to pediatrics are being developed: SPIRIT-Children (SPIRIT-C) and CONSORT-Children (CONSORT-C).

**Methods:**

The development of SPIRIT-C/CONSORT-C will be informed by the Enhancing the Quality and Transparency of Health Research Quality (EQUATOR) method for reporting guideline development in the following stages: (1) generation of a preliminary list of candidate items, informed by (a) items developed during initial development efforts and child relevant items from recent published SPIRIT and CONSORT extensions; (b) two systematic reviews and environmental scan of the literature; (c) workshops with young people; (2) an international Delphi study, where a wide range of panelists will vote on the inclusion or exclusion of candidate items on a nine-point Likert scale; (3) a consensus meeting to discuss items that have not reached consensus in the Delphi study and to “lock” the checklist items; (4) pilot testing of items and definitions to ensure that they are understandable, useful, and applicable; and (5) a final project meeting to discuss each item in the context of pilot test results. Key partners, including young people (ages 12–24 years) and family caregivers (e.g., parents) with lived experiences with pediatric clinical trials, and individuals with expertise and involvement in pediatric trials will be involved throughout the project. SPIRIT-C/CONSORT-C will be disseminated through publications, academic conferences, and endorsement by pediatric journals and relevant research networks and organizations.

**Discussion:**

SPIRIT/CONSORT-C may serve as resources to facilitate comprehensive reporting needed to understand pediatric clinical trial protocols and reports, which may improve transparency within pediatric clinical trials and reduce research waste.

**Trial Registration:**

The development of these reporting guidelines is registered with the EQUATOR Network: SPIRIT-Children (https://www.equator-network.org/library/reporting-guidelines-under-development/reporting-guidelines-under-development-for-clinical-trials-protocols/#35) and CONSORT-Children (https://www.equator-network.org/library/reporting-guidelines-under-development/reporting-guidelines-under-development-for-clinical-trials/#CHILD).

## Background

Well-designed, properly conducted and reported randomized clinical trials (RCTs) are needed to provide reliable evidence for advancing medical interventions and improving health outcomes in children and youth. However, several challenges and barriers prevent high-quality pediatric clinical trials from being conducted, such as difficulties in recruiting sufficient trial participants, research ethics issues, and limited funding [1–3]. As a result, there is a paucity of randomized clinical trials conducted in children, maintaining a dearth of pediatric-specific evidence [2]. In addition, when pediatric trials are not adequately designed, conducted, or their reporting is deficient, users of the evidence are unable to critically appraise and interpret findings with the full context, replicate results, and make critical healthcare decisions. While insufficient reporting is a pervasive issue in medical research in general [4–7], the negative effects of poor reporting are compounded further in pediatrics due to the limited opportunities to conduct pediatric trials and contribute to a biased record of available research results and lagged progress in the field with missed opportunities to improve patient care [1, 8].

To date, completed and published pediatric clinical trial protocols and reports are often inadequately reported and missing key elements [9–11]. Specifics on whether a systematic review was conducted [9], details on the primary outcomes [12–14], outcomes in relation to children and youth’s age and development [9], measurement properties of outcome measures [12], description of the control arm interventions [15, 16], and various social determinants of health, including race and ethnicity of participants [17], sexual orientation and gender identity [18], preferred language [18], and socioeconomic factors [18], are often unreported. As of 2023, there is still a lack of reporting standards specific to pediatric clinical trials, with a recent systematic review only identifying four published pediatric-research-specific standards, none of which were endorsed by pediatric journals [19]. Additionally, these standards were only applicable to certain pediatric fields (e.g., dentistry, early childhood development), and uptake of these standards remains low [19]. Evidently, there is still an unmet need for pediatric specific guidance to improve the understandability, interpretability, and utility of pediatric clinical trial protocols and reports.

While the Standard Protocol Items: Recommendations for Interventional Trials (SPIRIT) for trial protocols [20] and Consolidated Standards of Reporting Trials (CONSORT) [21] for trial reports will be updated in early 2024 to include the most recent developments in trial (protocol) transparency and publishing [22, 23], neither address the reporting of key details unique to conducting research in children and youth like age appropriate dosing and routing of drug interventions [24], developmentally appropriate (primary) outcome selection and measurement [25], sample size calculations [26, 27], issues surrounding consent and assent [3, 28–32], and the need to consider heterogeneity of treatment effects in different age subgroups within pediatrics [3, 33]. Thus, SPIRIT/CONSORT 2024 do not account for recent advances in the field of pediatric clinical trials nor involved pediatric trial participants or their families to ensure that reporting items that are important to their decision-making are captured.

Within pediatric research, there has been an increase in efforts to involve patients and families as partners in research, such as in the formulation of research questions to align with patient priorities, the design of research studies including the selection of outcomes and outcome measurement instruments, and the development of study materials [34]. Evidence has shown that meaningful patient and family involvement leads to the design and conduct of research that is relevant and important to those who are ultimately affected by the results, and this involvement leads to empowering patients, families, and researchers [34, 35]. As public mistrust in science and research continues to grow, recently fueled by rapid publication of misinformation and poorly designed studies during the COVID-19 pandemic [7, 36], there is an urgent need for standards and guidelines that promote transparency in child health research, developed with input from children, youth, and families. Comprehensive reporting and publication of pediatric trial results is critically needed, so that available findings can be utilized by clinicians, patients, and caregivers, be integrated into clinical practice, and inform shared clinical decision-making around treatment options that benefit patients.

Considering the evolving landscape in trial reporting and the push for transparency in pediatric research, the development of children and youth trial extensions to SPIRIT 2024 and CONSORT 2024 is timely [37]. We aim to develop the SPIRIT-C and CONSORT-C extensions to (1) align with the updated SPIRIT/CONSORT 2023 guidelines; (2) account for updates and advances made in the field of pediatric clinical trials since the initial pediatric-specific items from earlier efforts (“base checklist”); and (3) involve varied partner groups, including young people and family caregivers throughout the development process to ensure capture of key reporting items that provide the necessary context for readers to interpret, replicate, and synthesize pediatric trial findings. This protocol outlines the development process of the SPIRIT-C and CONSORT-C reporting guideline extensions, which first builds upon past initial efforts, and expands further to incorporate recent advancements in research to develop and finalize reporting guideline extensions applicable to all pediatric controlled clinical trials.

## Methods/design

### Aim and objectives

**Fig. 1:**
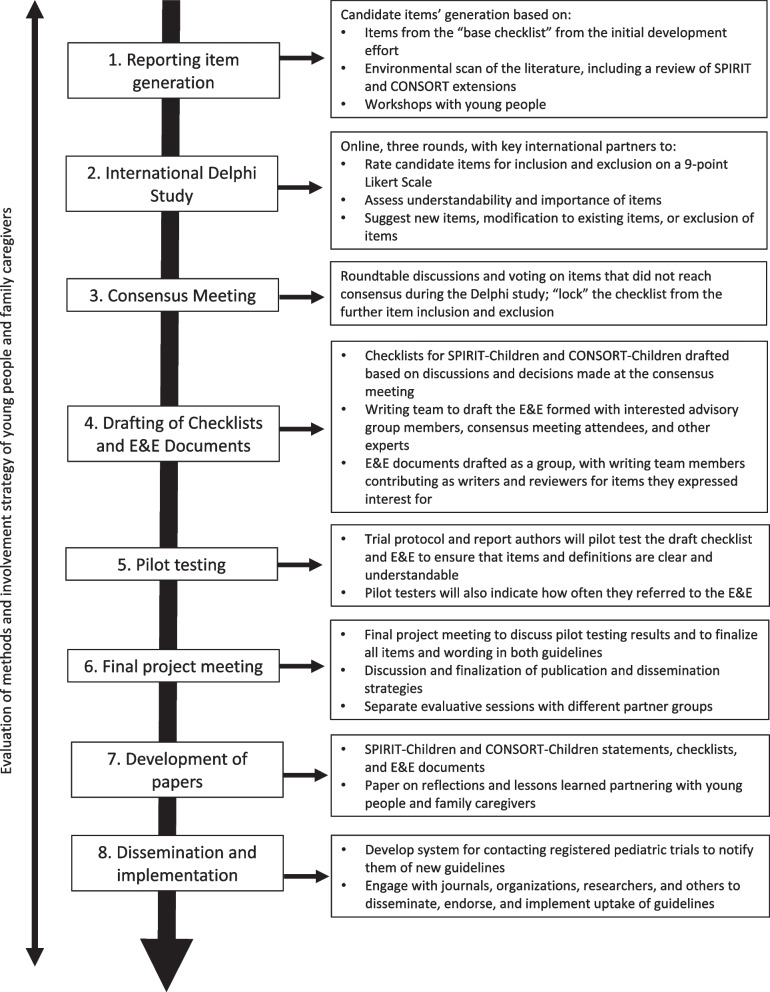
SPIRIT-Children and CONSORT-Children development process. E&E, Explanation and Elaboration

The stages adopted for developing the SPIRIT/CONSORT-C extensions are informed by the Enhancing the Quality and Transparency of Health Research Quality (EQUATOR) Network’s guideline on developing reporting guidelines [38], updates of other reporting guideline extensions [23, 39–43], and with consideration of identified methodological limitations in the development of reporting guidelines [44] (Fig. 1). Additionally, novel methods not yet formally integrated in the EQUATOR guidance will be incorporated, such as the inclusion of patient/caregiver members throughout the process, as was done successfully in recently developed reporting guidelines [45–47]. The overall objectives of this initiative include:

1. To identify and prioritize reporting items that will improve reporting comprehensiveness in pediatric clinical trial protocols and final reports;
2. To reach consensus on a minimum set of core pediatric reporting items applicable to all pediatric controlled clinical trials with a diverse partner group, including young people, family caregivers, and experts around the world; and
3. To implement innovative knowledge translation and dissemination strategies to maximize uptake and adherence.

We registered our intention to develop the SPIRIT-C and CONSORT-C extensions on the EQUATOR Network website in March 2023 [48, 49]. An Open Science Framework (OSF) Page (https://osf.io/bka4e/) was also created for this initiative [50].

### SPIRIT-C and CONSORT-C development group

#### Core project team

Oversight of the SPIRIT/CONSORT-C development will be provided by the SPIRIT/CONSORT-C core project team. The core project team will lead, design, and organize all stages of the development. Members will not participate in the Delphi study as panelists but will have the capacity to make final executive decisions and hold executive power to override consensus meeting decisions, if need be.

The core project team comprises of six individuals, the authors of this protocol. They collectively have expertise in child health research, pediatric rare genetic diseases, trial methodology, trial outcome selection, measurement, and reporting, patient engagement, and reporting guideline development and includes executive group members of the SPIRIT 2013/CONSORT 2010 and SPIRIT/CONSORT 2023 development groups. One core team member is a patient engagement expert (MS) and citizen leader who has collaborated on numerous research projects as a patient partner, has lived experience of participating in a pediatric clinical trial, and has extensive experience in engaging youth and patient partners to collaborate and work alongside researchers. The patient engagement expert will continue to be actively involved in decision-making throughout the entire duration of the project and will co-lead and advise on patient engagement initiatives throughout the development process.

#### International advisory group

The international advisory group will comprise of pediatric trial network representatives and experts in specific child health trial areas, who will support the core project team. They include pediatric clinical trialists, pediatrician-scientists, methodologists, reporting guideline developers, child health core outcome set developers, biostatisticians, epidemiologists, medical journal editors, systematic reviews and pediatric clinical trials evidence synthesis authors, and others. Through their existing collaborations, they will be asked to identify additional patient/public partners, pediatric networks, and others with relevant expertise who may be invited to join the project. International advisory group members will be involved and give feedback on the project materials and progress throughout the project, be Delphi study panelists, attend the consensus meeting, pilot the draft checklists, contribute to the Explanation and Elaboration (E&E) documents, and join the final project meeting. They will provide feedback on the drafts of the checklists, reporting guideline statements and related E&E documents, and facilitate dissemination efforts of the final reporting guidelines.

#### Other partners—Delphi panelists, consensus meeting attendees, pilot testers

The core project team and the international advisory group will identify additional experts and partners, which include, but are not limited to regulators, research funders, and journal peer reviewers. These additional identified partners will be invited to contribute to various stages of the project, such as the Delphi study, pilot testing, and in dissemination efforts. Core project team and international advisory group members will circulate a project information leaflet and link to an online interest form developed using the Research Electronic Data Capture (REDCap) system [51] to their colleagues, personal networks, and pediatric trial networks they are associated with. The online interest form will ask respondents to provide a brief overview of their background and experiences and express their interest in participating in the development process as a Delphi panelist, consensus meeting attendee, pilot tester, or other role. Partners will also be identified at academic conferences where the members of the core project team and international advisory group are presenting on the project to raise awareness. Afterwards, those who have expressed interest and have the relevant experiences and background will be contacted by the core project team regarding their involvement and specific contributions.

#### Young people (ages 12–24 years) and family caregivers

During the early development efforts of SPIRIT/CONSORT-C back in 2014, patient/caregiver partners were not engaged in the identification, synthesis, and finalization of reporting items. In our current efforts, we aspire for these checklists to be relevant and useful for children, youth, and their families as end-users of clinical trial protocols and trial results. Therefore, we will involve them in multiple ways: (1) as advisors involved in several steps of the project; (2) for young people, as contributors to the Young Persons Reporting Guideline workshops; and (3) for family caregivers and young people ages 19–24, the Delphi study. We will involve (1) young people (ages 12–24) with (a) lived experiences of being a pediatric clinical trial participant and/or (b) who read and use trial results to inform their healthcare decision-making (as research end-users) and/or (c) have experience collaborating and advising researchers on a research team and (2) family caregivers (e.g., parents) who (a) have a child that participate(d) in a pediatric clinical trial and/or (b) use trial results for decisions and/or shared decision-making regarding their child’s condition. The selected age range is based on the World Health Organization (WHO)’s definition of young people (ages 10–24) [52] and the age range of the members of various international Young Persons Advisory Groups (YPAG) involved in this project (described below); the youngest is 12 years old in these groups. This age range will allow us to include the perspectives of young people with current and recent experience of participating in a pediatric clinical trial. While most young people will be involved in the YPRG workshops, those between the ages of 19 and 24 will also be invited to contribute to the Delphi study. Young people and family caregivers will be invited to be involved in the development of SPIRIT/CONSORT-C in varying capacities with recognition of their interest, perspectives, and skills, so that established partnerships are meaningful, appropriate, and sustainable for everyone involved [53].

#### Involvement of young people

Young people from the Maternal Infant Child and Youth Research Network (MICYRN) KidsCan Young Persons Advisory Group (YPAG, https://www.micyrn.ca/ypag) were consulted on how they think youth would want to be involved in the project, what would make youth want to be involved, and what would help them be involved (e.g., information, training). Additionally, a Youth Involvement International Steering Committee was formed with members of the core project team (MS, AB, MO) and YPAG facilitators from the United Kingdom (UK) and Europe. The facilitators were consulted on how to meaningfully involve their YPAGs in this project, based on their expertise of working with their respective groups under the umbrella of eYPAGnet (European Young Persons Advisory Groups Network, https://eypagnet.eu/). Based on the advice from the KidsCan YPAG and eYPAGNet facilitators, young people will have varying options for involvement, with most young people involved in the project based on a “hub-and-spoke” model (focus group format and other adapted methodologies to the age of the participants: children, young people, and parents). We adopted a hub-and-spoke model to offer flexibility and accommodate different levels of involvement, and to reach a broad, international group of young people with varied experiences.

A Pan-Canadian Youth Advisory Group (YAG) consisting of 5 to 7 young people will be formed. Members of this YAG will be involved (i.e., partner) in the project from the beginning to the end and will meet to advise on various project materials, workshop content, and knowledge translation strategies. YAG (“hub”) meetings will be led by core project team members. Discussions and decisions made during the YAG meetings will inform planning of project stages, such as the Young Persons Reporting Guideline (YPRG) workshops, and deliverables.

Young people outside of the YAG will also be involved as contributors to the YPRG workshops. A series of two workshops will be conducted with existing YPAGs (“spokes”) based in the UK, Europe, and Canada. Each workshop will be conducted in each YPAG, in their native language, and led by their respective YPAG facilitator to foster active involvement of young people within their groups, as they are the most experienced in working with their YPAG. Workshop content will include project onboarding, training, and discussions of potential items for the SPIRIT-C and CONSORT-C checklists. The development of workshop materials will be led by the core project team with advice from the YAG to standardize workshop activities, so that questions and items for feedback discussed with youth in different YPAGs remain consistent. YPAG facilitators will also collaboratively work with the core project team on these materials and will adapt the materials accordingly for their YPAGs. For example, workshops will be conducted in the language of the existing YPAGs. Subsequently, feedback obtained from YPAGs will be translated to English and shared with the “hub” (i.e., core project team). Feedback received from each YPAG after every workshop will be reviewed by the hub and integrated into the project based on the project stage that the workshop was relevant to.

The existing YPAGs in the UK and Europe will decide on compensation for young people within their groups to recognize their time and contributions in accordance with their own group’s guidelines. For the Canadian YAG and YPRG workshop attendees, compensation will be provided based on the Canadian Institutes of Health Research (CIHR) Strategy for Patient Oriented Research (SPOR) guidelines [54]. Aside from monetary compensation, the YAG will be consulted on other ways young people can be appreciated for their involvement (e.g., volunteer credits, letters of reference).

#### Involvement of family caregivers

We aim to involve an international group of caregivers (e.g., parents) of children who participate(d) in a pediatric clinical trial and/or with experience using trial results for healthcare decision-making for their child. A Family Caregiver Advisory Group (FCAG) comprising of 5 to 7 caregivers will be formed. The FCAG will regularly meet from the beginning of the project to discuss and plan various project materials, content for various project stages (e.g., Delphi, consensus meeting, E&E writing, final project meeting), and knowledge translation strategies. Similar to the YAG, feedback from the FCAG will be reviewed centrally by the hub and will inform project deliverables. Family caregivers will have the opportunity to be involved at a consultation, involvement, and collaborative level, as described by Manafo et al. [55]. Those who fulfill the criteria of authorship will be invited to be co-authors on publications from the project.

Additional family caregivers will be involved as Delphi contributors. They will be identified through existing pediatric (subspeciality or disease focused) research networks. Our approach to engaging family caregivers outside of the FCAG will be adapted from the strategy co-developed by the patient engagement expert and the PRISMA-COSMIN for OMIs project team, which has undergone rigorous evaluation to identify barriers, facilitators, and lessons learned in engaging patient/ public members in the development of a recent reporting guideline, PRISMA-COSMIN for OMIs [47].

Training sessions will be held prior to each project stage to help family caregivers to be prepared and contribute meaningfully, to have the opportunity to ask questions, and to set expectations by clearly defining their roles for that specific project stage. Similarly to the young people, compensation for family caregiver’s time and efforts is planned according to CIHR SPOR guidelines [54].

#### Evaluation

We will assess our approach to engaging with young people and family caregivers throughout the entire project, including evaluating the impact of young people and family caregivers. In doing so, we will use evaluation tools including the Public Involvement Impact Assessment Framework (PiiAF) to consider how young people and family caregivers were involved throughout the process and to assess the impact of their involvement (e.g., generating items, improving readability, knowledge translation strategy) on the development of the final SPIRIT/CONSORT-C checklists [56]. Throughout the project, the impact of young people and family caregivers on each project stage will be recorded.

Young people and family caregivers will be asked to complete modified versions of the Public and Patient Engagement Evaluation Tool (PPEET) [57] and the Patient Engagement in Research Scale (PEIRS) [58] both tailored to the development of SPIRIT-Cand CONSORT-C, as there are few evaluation tools that have been designed for evaluating patient and public involvement in methodological projects. Debrief sessions after various project stages will supplement the results from PPEET and PEIRS and allow for the collection of qualitative feedback on the engagement experience.

### Stage 1: Reporting item generation

The final SPIRIT/CONSORT-C reporting guideline extensions will comprise of a minimum set of items that are applicable and important to report in all pediatric controlled clinical trial protocols and reports. A base checklist containing items generated from the initial development effort of the SPIRIT/CONSORT-C extensions will serve as a starting point. An environmental scan of the literature will be conducted to search for new articles and guidance published since the systematic review published in 2015 [59]. Documents pertaining to reporting in pediatric clinical trial protocols and reports, those that mention information that should be reported in pediatric clinical trial protocols or reports, or recommendations for reporting will be eligible. Information garnered from these sources will be compiled and assessed to generate additional potential items for inclusion in the base checklist. As part of this environmental scan, all SPIRIT and CONSORT reporting guideline extensions available as of November 2023 will also be reviewed to identify additional items that are potentially relevant for inclusion in a minimum set of core items to be reported in pediatric clinical trial protocols and reports.

Young Persons Reporting Guideline workshops will be conducted to generate items that they deem important for trialists to report on in their trial protocols and reports. Previous work done by our team in developing CommuniKIDS [60], a plain language, trial results summary template, where youth and parent advisors provided their input on what they would like to see in a trial results communication template, will serve as a discussion starting point. Ideas generated during these workshops will be taken on board as potential additional reporting items for the final SPIRIT/CONSORT-C checklists.

Items from the base checklist will be reviewed in the context of the new SPIRIT and CONSORT 2023 reporting guidelines. All items will be reassessed by the core project team in the context of the findings from the environmental scan of the literature and the Young Persons Reporting Guideline workshops to ensure that they are still relevant. Items generated from the environmental scan of literature, review of existing reporting guideline extension items, and YPRG workshops will be reviewed and integrated with the base checklist to generate a comprehensive list of “candidate” items for inclusion in the checklists. All preliminary “candidate” items will be placed in the appropriate sections of the SPIRIT and CONSORT 2023 reporting guidelines and clustered based on the item’s topic. Delphi panelists will be able to see if the item was generated from the Young Persons Reporting Guideline workshops. This list of candidate items will be reviewed by the core project team and will be included in the Delphi study.

### Stage 2: International Delphi Study

A three-round, international Delphi study will be conducted online. A web-based questionnaire will be developed using REDCap [51]. The Delphi method is a structured process in which individual, confidential feedback from multiple partners can be gathered in an organized manner to reach consensus [61, 62]. Candidate reporting items generated in the previous stage will be evaluated during the Delphi by panelists. The Delphi study will result in a refined list of items that will be assessed at the consensus meeting, informing which items should be included, modified, or excluded from the two final guidelines based on a priori criteria as described below.

#### Identification and recruitment of Delphi participants

We will invite a diverse range of key international partners to complete the Delphi study as panelists. There will be no geographical restrictions of where panelists are from, and the Delphi study will be conducted in English. The core project team and the international advisory group will identify individuals, networks, and organizations through their professional contacts, affiliations, and networks. We aim to include individuals with involvement and expertise in pediatric clinical trials and child health research (e.g., pediatric clinical trialists, child health researchers, trial methodologists, clinician-scientists, systematic reviewers), members of international pediatric networks including but not limited to the Maternal Infant Child and Youth Research Network (MICYRN), Ontario Child Health Support Unit (OCHSU), INFORM RARE, Pediatric Inpatient Research Network (PIRN), Pediatric Emergency Research Canada (PERC), Pediatric Trials Network (PTN), TARGet! Kids, Increasing Capacity for Maternal and Paediatric Clinical Trials (IMPaCT), conect4children (C4C)), and interested family caregivers and young people (ages 19–24). Those who have completed other Delphi studies as panelists and authors from relevant documents and articles identified through the environmental scan of the literature will also be invited.

Potential Delphi panelists will be formally invited through e-mail, where they will be able to access a panelist registration form online; all invitation e-mails will also have a forwarding option for invitees to identify and share the invitation with other potential Delphi panelists who may be interested. The registration form will collect informed consent, in addition to basic demographic information (e.g., job title, primary affiliation/organization, country of workplace, level of education, relevant work experience) to evaluate the diverse representation of partners. Demographic questions will be adapted for young people and caregivers (e.g., remove work experience, level of education). Additionally, the registration form will ask respondents to indicate their relevant experience and involvement in the following research activities: designing, conducting, or providing oversight to a pediatric clinical trial, including statistical planning; authoring or reviewing pediatric clinical trial protocols or reports; conducting systematic reviews or evidence synthesis of pediatric clinical trials. Respondents will be asked whether they have contributed to pediatric trial protocols/reports and how frequently they are consumers of clinical trial reports or systematic reviews of trials to inform their decision-making. Questions for young people and caregivers will be adapted to ensure that they meet the criteria established. Individuals who complete the registration form that do not have any relevant experiences will not be eligible to be a Delphi panelist. Eligible individuals interested in contributing as a panelist will be asked to voluntarily commit to complete all rounds of the Delphi study.

All family caregivers and young people (ages 19–24) who have indicated interest in being a Delphi panelist will attend a training session prior to the launch of the Delphi study. This session will be conducted to provide an opportunity for them to ask the project team questions, to facilitate their understanding of the objectives of the project and importance of transparent reporting in trial protocol and reports, and to provide them with an overview of the Delphi study to help them feel prepared to partake in the process.

Although there are no strict guidelines with regards to how many people should participate in a Delphi study [63, 64], we aim to directly invite 200 individuals to complete the Delphi study, as per similar studies conducted previously [23, 45–47]. Our reach will be supplemented through snowballing through the *international advisory group’s* networks and contacts; Delphi registrants will be invited to share their invitation and suggest colleagues to register as a Delphi panelist. Our minimum number of Delphi panelists is 30 [47, 62], with a target of 200 panelists (15 family caregivers, 5 young people (ages 19–24), and 180 experts). Depending on the number of people who accept their invitation to complete the first round of the Delphi study, the recruitment strategy will be modified to increase the number of individuals or to improve diversity in partner groups represented.

#### Delphi study procedure

After registration, Delphi panelists will receive further information on the project objectives and the Delphi process. Each Delphi round will be open for around 3 weeks; a reminder will be sent to all registered panelists 1 week after the start of the round. We will ask panelists to keep four considerations in mind while reviewing candidate items for inclusion, specifically, whether the item (1) will foster transparent, comprehensive reporting in pediatric clinical trial protocols or reports; (2) will facilitate the assessment of a trial’s quality and applicability; (3) is relevant to most pediatric clinical trials; and (4) should be included in a minimum set of items that should be reported in all pediatric controlled clinical trial protocols (SPIRIT-C) and trial reports (CONSORT-C). All responses will remain confidential throughout the duration of the Delphi study and during the analysis. Core project team members will be able to access the identity of respondents, link responses across rounds, and send targeted reminders.

The survey, invitation text, and associated materials will be pilot tested by members of the core project team and members of the international advisory group who have elected not to complete the Delphi study as panelists. After pilot testing, all materials will be revised based on the feedback received.

#### Round 1

Delphi panelists will be asked to review all candidate items generated in stage 1 for inclusion in SPIRIT-C and CONSORT-C. Panelists will be able to see if an item is new (i.e., not in the base checklist), existing (i.e., from the base checklist), or modified for use in pediatric trials (i.e., textually revised existing item). For all new and modified items, panelists will be asked to rate the item on a 9-point Likert scale, with ratings of 1–3 indicating limited importance for inclusion, 4–6 indicating important but not critical for inclusion, and 7–9 indicating critical for inclusion. The 9-point Likert scale was selected as it was successfully used in multiple previous studies [45, 46, 65–67]; criteria for inclusion and exclusion using this scale are described most frequently in the literature [64, 68, 69]. Panelists will be able to see which items come from the base checklist; these may be voted out.

Panelists will also be given an option, “I'm opting out,” which can be used by panelists for items they feel that they do not wish to rate. Along with the rating options, a free-text box will be available for each item, where panelists will be encouraged to elaborate on their selected rating for the item, suggest revisions to the wording of the item or definitions to enhance clarity, indicate overlap with other items, or suggest new items. At the end of the round 1 survey, there will be another free-text box where panelists will be able to provide any other comments and suggestions or suggest new items after reviewing all potential candidate items. After round 1, an optional check-in session will be held with the family caregivers and young people who contributed as panelists.

#### Rounds 2 and 3

The same Delphi panelists that completed round 1 will be invited to round 2. A feedback report with a summary of anonymized aggregate results from all Delphi panelist’s ratings for items from round 1 will be sent to all panelists. When they access the round 2 survey, for each item, they will be presented with their original rating from round 1, in addition to an anonymized summary of results from other panelists for the item. The summary of results will provide panelists with the distribution of ratings from others, in addition to the textual suggestions and comments received for the item. For items that did not reach a high level of agreement for inclusion in round 1, panelists will be asked to rate each item again, with consideration of their original rating and the summary of results. A free-text box will be available for panelists to elaborate on their selected rating for the item, suggest revisions to the wording of the item or definitions to enhance clarity, or indicate overlap with other items; however, they will not be asked to suggest new items. The process for round 3 will be similar to round 2.

#### Delphi study analysis

Once all rounds are completed, the results will be analyzed to determine if items have reached consensus for inclusion, exclusion, or require further discussion at the consensus meeting. Summarized findings of the ratings will be compiled in a final feedback report, along with qualitative comments and suggestions from panelists. Items that have been assessed at least twice and have reached ≥ 70% consensus for exclusion based on scores of 1–3 (“limited importance”) and < 15% of panelists rated it with scores 7–9 (“critical for inclusion”) will be excluded and no longer considered or discussed at the consensus meeting. Items that have been assessed at least twice, reached ≥ 70% consensus for inclusion based on scores 7–9, and < 15% of panelists rated it as score 1–3 for inclusion will be included and not discussed at the consensus meeting. Though, at present, there is no single consensus criterion that has been demonstrated to be superior to others [68], these consensus thresholds are based on previous studies [45, 46, 65–67]. All other items, where no consensus was reached, where the item was assessed only once, or where panelists have suggested changes to wording that need to be discussed, will be reviewed by the core project team and brought forward for discussion at the consensus meeting.

### Stage 3: Consensus meeting

A half-day, virtual consensus meeting will be hosted using Zoom. The objective of this meeting is to reach expert group consensus on items that have not reached consensus for inclusion and exclusion, as described above, to “lock in” items that will be included in SPIRIT-C and CONSORT-C extensions. Members of the core project team and the international advisory group, individuals from groups represented on the Delphi survey, and Delphi panelists with relevant expertise will be invited. FCAG members who express interest in attending the consensus meeting will also be invited. We aim to have at least 20–25 expert attendees from diverse backgrounds and relevant experiences attend the consensus meeting; purposive sampling and invitations of individuals with relevant expertise and backgrounds may be done if sufficient representation and attendance is not achieved. The meeting will be recorded, and the meeting chat will be captured to facilitate notetaking.

Consensus meeting materials, including the agenda and the feedback report from the Delphi study, will be prepared by the core project team and shared prior to the meeting to all attendees. At the meeting, an overview of the Delphi study results will be presented, with specific focus on items that have not yet reached consensus. Meeting attendees will discuss the findings for these items, discuss wording, and vote anonymously on the inclusion or exclusion of items that do not yet have consensus for inclusion. Voting choices will include three options: “Include”, “Exclude”, or “Abstain”; at least 70% is needed for consensus to be reached. If a consensus is not reached after the first vote, moderated discussions will continue until consensus is reached or time has run out. In the situation that an item did not reach consensus at the end of the meeting, the core project team will make the executive decision on its inclusion or exclusion.

### Stage 4: Drafting of checklists and Explanation and Elaboration (E&E) documents

Drafting of the final checklists will occur after the consensus meeting. Taking the discussions from the consensus meeting into account, the core project team will review the wording of each item and draft both the SPIRIT-C and CONSORT-C checklist.

At the end of the consensus meeting, plans on writing the E&E documents through the formation of a E&E writing group will be introduced and discussed. Though the core project team will lead the drafting of the E&E documents, we will adopt a group writing process as was done for similar projects [23, 70, 71]. After the consensus meeting, those who attended and are interested in joining the E&E writing group will be able to sign-up and select the roles in which they would like to contribute—as a writer, to draft E&E text(s) for items that are interest to them, as a reviewer, to review drafted E&E texts for reporting items of interest to them, or both. Reporting items will be clustered into sections based on the topic the item pertains to; therefore, during the sign-up process, writing group members will be asked to indicate their preferences for which items they would like to contribute or review text for. The core project team will review registrants and will assign individuals based on their preferred roles and items. In the situation that there are items that have an insufficient number of writers or reviewers interested, core project team members will supplement and write and review those items. Each item will have at least two writers and two reviewers. Multiple core project team members will review all written contributions from writing group members to verify that the E&E text for each item is clear, appropriate, and understandable. Those in the writing group who meet criteria for authorship as per the International Committee of Medical Journal Editors (ICMJE) criteria will be included as co-authors [72].

### Stage 5: Pilot testing

Authors of pediatric trial protocols, pediatric trial reports, peer reviewers, and others involved in drafting and revising pediatric trial protocols and reports will be invited to take part in pilot testing. We will send emails to pediatric clinical trialists who have registered a pediatric clinical trial in the last 6 months, as identified through ClinicalTrials.gov. Invited individuals will be eligible to pilot test if they are in the process of drafting or submitting a pediatric trial protocol and/or report during the piloting phase. We aim to include a minimum of 50 pilot testers.

The online pilot testing survey will be prepared on REDCap [51]. Pilot testers will be sent a link to the survey and will be provided with the checklists and E&E documents. They will be asked to use the draft checklist in relation to their trial protocol or report and refer to the E&E as need be during the process. The pilot testing survey will ask respondents to rate the usability of the checklist (i.e., ease of use, time it takes to use the checklists, likelihood of using checklists) and provide feedback on the clarity and understandability of the items. For items that pilot testers indicate are unclear or difficult to understand, there will be a free-text box where they will be able to elaborate on their reasons. Pilot testers will not be able to suggest any new items at this point. Pilot testing feedback will be reviewed and brought forward for discussion at the final project meeting. We aim to include at least 50 pilot testers.

### Stage 6: Final project meeting

After pilot testing, a 2-day final project meeting will be conducted virtually. The objective of this meeting is to finalize the items in the context of the pilot testing results and discuss publication and dissemination strategies. Modifications may be made to the items in consideration of feedback received from pilot testing. The final project meeting will comprise of multiple sessions dedicated to different partner groups (e.g., journal editors, family caregivers, pilot testers) and by topic sections in the checklists. All items and sections will be reviewed in the context of the pilot testing results by the core project team. Publication strategies and dissemination plans will also be discussed.

### Stage 7: Development of papers

The core project team will finalize the checklists after the final project meeting and will lead the writing of the SPIRIT-C and CONSORT-C statement papers. A “statement” paper describing the development process and highlighting the new checklists will be prepared for both SPIRIT-C and CONSORT-C. The E&E documents will also be prepared for publication, as these documents describe each new item’s rationale along with good reporting examples.

Evaluations and feedback from Advisory Group Meetings, Young Persons Reporting Guideline workshops, and young people and family caregivers will be reviewed and assessed, and a paper describing the work done with the advisors, young people, and family caregivers will be drafted. This paper will discuss and reflect on involvement of young people and family caregivers and the various lessons learned to serve as a resource for those seeking to conduct methodological work with young people and family members. Those who meet ICMJE authorship criteria will be co-authors on the paper.

All papers will be circulated to relevant partners who were involved in the development and consensus process of SPIRIT-C and CONSORT-C for review and revision. Once all feedback is incorporated, the papers will be submitted for publication.

### Stage 8: Dissemination and implementation

All publications will be submitted and published “open access”, under a Creative Commons license (CC BY). We also plan to develop tip sheets for each item describing the item and providing examples of reporting. These tip sheets will be available online. Downloadable, fillable versions of the checklists will be posted on the SPIRIT and CONSORT websites, respectively. The EQUATOR Network Database will also be updated with relevant links to all publications, checklists, and tip sheets produced from this project. We will also reach out to pediatric journals with the intention of having these journals endorse a requirement for authors to use the SPIRIT/CONSORT-C guidelines when submitting pediatric trial protocols and reports. Adherence to reporting guidelines are higher in articles that are published in journals that endorse their use [73–76]; in addition, lack of endorsement by pediatric journals for currently available pediatric reporting standards may be a factor in low uptake [19]. Therefore, this is likely a critical step in increasing awareness, uptake, and adherence to the newly developed SPIRIT/CONSORT-C to improve reporting comprehensiveness in future trial protocols and reports. However, endorsement from journals is not sufficient, particularly for trial protocols, as adherence to SPIRIT guidelines is still suboptimal [77]. While trial protocols are often reviewed at early stages by funders, Institutional Review Boards, and regulatory bodies, these early review efforts need to be more transparent, connected, and require structured guidelines for peer review [78]. For pediatric trial protocols, the use of SPIRIT-C in these early peer review stages, and not just at the stage of submission to a journal, may improve the transparency of the trials’ design and planned conduct. Users will also be able to find all materials and resources, which will be freely available, on the SPIRIT-C and CONSORT-C site at https://lab.research.sickkids.ca/enrich/. Project materials will be made publicly available on the Open Science Framework at https://osf.io/bka4e/ [50].

Where possible, the results of the project will be presented at academic conferences at the local, national, and international level. As part of the efforts to increase implementation of and adherence to SPIRIT-C and CONSORT-C, we will also search various clinical trial registries (e.g., ClinicalTrials.gov, Health Canada Clinical Trial Database, EU Clinical Trials Register, Australian New Zealand Clinical Trials Registry) to find registered pediatric trials that are currently active and will reach out to registered investigators of these trials to alert them of the new reporting guidelines and to encourage them to use them in the preparation of their trial reports and future trial protocols.

## Discussion

The SPIRIT-C and CONSORT-C guidelines will be developed to provide guidance and standardization on the minimum set of information that should be reported in pediatric trial protocols and reports. The evaluation and incorporation of key items from the initial efforts, items from existing SPIRIT and CONSORT extensions, and other newly identified items from the environmental literature scan and Young Persons Reporting Guideline workshops will result in guidance that is all-encompassing for all pediatric controlled trial protocols and reports. Mindful of several criticisms of reporting guidelines, including that more than one reporting checklist may be applicable to any given study and it is both time consuming and burdensome for investigators to find and implement all applicable guidelines [79], SPIRIT/CONSORT-C will streamline trial authors’ reporting efforts. Specifically, when more than one reporting standard is applicable, authors are encouraged to combine reporting items from these other relevant extensions with the child-specific items in SPIRIT/CONSORT-C. Importantly, what the journals are prepared to accept is crucial. This is one of the main reasons for engaging journal editors throughout the development process. Investigators designing, conducting, and reporting pediatric clinical trials will be able to refer to and use the harmonized SPIRIT-C and CONSORT-C checklists for both their pediatric trial protocols and reports. This approach will reduce burden on authors, journal editors, and peer reviewers.

While the inclusion of young people and family caregivers as a partner group in methodological research is still rare, efforts in involving patient/public partners have been increasing [45–47, 80]. As formal guidelines on how to form a true partnership in methodological research are not yet available, there may be challenges associated with identifying appropriate roles and expectations for everyone involved. However, as more experiences with patient/public partners are being published, it is evident that meaningfully partnering with patient/public partners in methodological work is possible and may maximize usefulness of the resulting best practice guidance [45–47, 80]. This project will be unique from others as this will be the first attempt in working with children and youth partners in the development of reporting guidelines. As they are directly impacted by the results of published pediatric trials, their involvement, perspective, and expertise are vital in ensuring the relevancy of the items in these planned guidelines.

We will also integrate virtual methods throughout the project. For example, many of the meetings will be held virtually, which opens the opportunity to work with a diverse group of international collaborators and pediatric networks, enabling the involvement partners from around the world. Both the consensus and final project meetings will be held virtually, replacing the traditional in-person consensus meeting structure. The adoption of electronic and virtual methods will be beneficial in the ability to invite and collaborate with many experts and groups internationally that may not have been feasible otherwise.

The SPIRIT-C and CONSORT-C guidelines may be helpful resources to improve the transparency and hence the replicability, understandability, and usability of pediatric trial protocols and reports, which may reduce ongoing waste in child health research. The methods for this development process are informed by evidence-informed and consensus-based methods. The project will involve many experts and key partner groups, which will contribute to its deliverables’ credibility, acceptance, and eventual uptake within the pediatric and child health research enterprise. Findings and results of the SPIRIT/CONSORT-C development efforts, i.e., the statement papers, reporting guideline checklists, and E&E documents, will be published in peer-reviewed articles. Given the paucity of pediatric-specific reporting guidelines and standards [19, 37], we anticipate that the SPIRIT/CONSORT-C guidelines will be much anticipated, be used widely, and improve the quality and reporting of future pediatric clinical trial protocols and reports.

### Trial status

Protocol version 1.0. Recruitment is not being conducted as part of this study.

## Data Availability

Project materials are publicly available on the Open Science Framework at https://osf.io/bka4e/.

## Abbreviations

CIHR: Canadian Institutes of Health Research
C4C: Conect4Children
COSMIN: COnsensus-based Standards for the selection of health Measurement Instruments
CONSORT: Consolidated Standards of Reporting Trials
CC BY: Creative Commons license
EQUATOR: Enhancing the Quality and Transparency of Health Research Quality
eYPAGnet: European Young Persons Advisory Groups Network
YAG: Youth Advisory Group
E&E: Explanation and Elaboration
GRIPP: Guidance for Reporting Involvement of Patients and the Public
IMPaCT: Increasing Capacity for Maternal and Paediatric Clinical Trials
ICMJE: International Committee of Medical Journal Editors
MICYRN: Maternal Infant Child and Youth Research Network
OCHSU: Ontario Child Health Support Unit
OMI: Outcome measurement instruments
PEIRS: Patient Engagement in Research Scale
PERC: Pediatric Emergency Research Canada
PIRN: Pediatric Inpatient Research Network
PTN: Pediatric Trials Network
PRISMA: Preferred Reporting of Items for Systematic reviews and Meta-Analyses
PPEET: Public and Patient Engagement Evaluation Tool
PiiAF: Public Involvement Impact Assessment Framework
REDCap: Research Electronic Data Capture
SPOR: Strategy for Patient Oriented Research
SPIRIT: Standard Protocol Items: Recommendations for Interventional Trials
UK: United Kingdom
YPAG: Young Persons Advisory Group
YPRG: Young Persons Reporting Guideline
